# Antitumor Effect of Periplocin in TRAIL-Resistant Human Hepatocellular Carcinoma Cells through Downregulation of IAPs

**DOI:** 10.1155/2013/958025

**Published:** 2013-01-01

**Authors:** Chieh-Fang Cheng, I-Huang Lu, Hsiang-Wen Tseng, Chung-Yuan Sun, Li-Tsen Lin, Zong-Keng Kuo, I-Horng Pan, Ching-Huai Ko

**Affiliations:** Biomedical Technology and Device Research Laboratories, Industrial Technology Research Institute, Hsinchu, Taiwan

## Abstract

Cortex periplocae is the dried root bark of *Periploca sepium* Bge., a traditional Chinese herb medicine. It contains high amounts of cardiac glycosides. Several cardiac glycosides have been reported to inhibit tumor growth or induce tumor cell apoptosis. We extracted and purified cortex periplocae and identified periplocin as the active ingredient that inhibited the growth of TNF-related apoptosis-inducing ligand-(TRAIL-) resistant hepatocellular carcinoma cells. The antitumor activity of periplocin was further increased by TRAIL cotreatment. Periplocin sensitized TRAIL-resistant HCC through the following two mechanisms. First, periplocin induced the expression of DR4 and FADD. Second, the cotreatment of TRAIL and periplocin suppressed several inhibitors of apoptosis (IAPs). Both mechanisms resulted in the activation of caspase 3, 8, and 9 and led to cell apoptosis. In addition, intraperitoneal injection (IP) of periplocin repressed the growth of hepatocellular carcinoma (HCC) in xenograft tumor model in mice. In summary, periplocin sensitized TRAIL-resistant HCC cells to TRAIL treatment and resulted in tumor cell apoptosis and the repression of tumor growth *in vivo*.

## 1. Introduction

Liver cancer is the fifth leading malignancy in men and the ninth in women worldwide [1]. The incidence of liver cancer is highly correlated to chronic local inflammation and cirrhosis. Therefore, factors that stimulate inflammation in liver, including alcohol uses, infection of hepatitis B and C viruses, and fatty liver diseases, are strongly correlated to the pathological progression of liver cancer.

Current treatments for liver cancer are limited. Hepatectomy can be used in early stage liver cancer patients with functional liver. Liver transplantation can help patients with damaged livers, but matching suitable donors is not easy. Although these surgical operations work well in early stage diseases, they are not helpful for patients with cancer cells spread out of the liver. Chemotherapy and internal radiation therapy are also options for liver cancer treatment. However, they may do damage to other tissues and organs as well. Targeted therapy is a more specific treatment for cancer. Sorafenib, a multireceptor kinase inhibitor with antiangiogenic activity, is the standard treatment for advanced hepatocellular carcinoma (HCC) that cannot be removed with surgery. Although it extends the median overall survival in patients with advanced HCC for nearly 3 months, sorafenib does not increase the median time to symptomatic progression in patients with advanced HCC [2]. Therefore, novel treatments for HCC are strongly in need. 

With the advancement of techniques in extraction, isolation, and recognition of compounds from plants, scientists started to search for antitumor components from herb medicine [3–6]. Cortex periplocae is the dried root bark of *Periploca sepium* Bge. It contains several cardiac glycosides and can be used in the treatment of various heart conditions. Recent studies also suggest that periplocin, a cardiac glycoside extracted from cortex periplocae, can inhibit cell growth in colon cancer cells and lung cancer cells [7, 8].

TNF-related apoptosis-inducing ligand (TRAIL) is a member of the tumor necrosis factor superfamily. It is also known as CD253 and APO-2L. TRAIL binds to the death receptors DR4 and DR5 and induces cell apoptosis [9–11]. Therefore, TRAIL is a potential candidate for cancer treatment [12]. In addition, phases 1 and 2 clinical trials for specific monoclonal antibodies against DR4 and DR5 have provided promising results [13].

Although TRAIL is a promising chemotherapeutic target for cancers, resistance to TRAIL-induced apoptosis has been reported in several different cancers, including colorectal cancer, breast cancer, liver cancer, and pancreatic cancer [14–17]. Several different mechanisms are proposed for TRAIL resistance [18]. Ways to overcome TRAIL resistance are still under investigation [19, 20]. We sought to investigate the effect of periplocin in sensitizing TRAIL-resistant HCC cell lines in this study.

## 2. Material and Methods

### 2.1. Cell Culture

HCC cell lines were purchased from different organizations. HA22T/VGH and Huh-7 were purchased from Bioresource Collection and Research Center (BCRC) in Taiwan. Huh-7 was purchased from Japanese Collection of Research Bioresources (JCRB). HA22T/VGH and Huh-7 were culture in DMEM (Gibco, Carlsbad, CA, USA) with 10% FBS and 100 mM nonessential amino acids (Gibco, Carlsbad, CA, USA).

### 2.2. Reagents

Recombinant human soluble TRAIL/APO2 ligand was purchased from ProSpec (Tany TechnoGene Ltd., Israel). Z-DEVD-FMK (CASP3 inhibitor), Z-IETD-FMK (CASP8 inhibitor), Z-LEHD-FMK (CASP9 inhibitor), and Z-VAD-FMK (pan CASP inhibitor) were purchased from R and D (Minneapolis, MN, USA). Monoclonal antihuman TRAIL R1 (TNFRSF10A,DR4)-Phycoerythrin antibody, antihuman TRAIL R3 (TNFRSF10C, DcR1)-Phycoerythrin antibody, and antihuman TRAIL R4 (TNFRSF10D, DcR2)-Phycoerythrin antibody were purchased from R and D (Minneapolis, MN, USA). PE antihuman TRAIL-R2 (TNFRSF10B, DR5) antibody was purchased from Biolegend. (San Diego, CA, USA) N-acetyl-cysteine (NAC) and DCHFDA were purchased from Sigma Chemical Co. (St. Louis, MO, USA). Hydrogen peroxide (H_2_O_2_) was purchased from MERCK (Whitehouse Station, NJ, USA).

### 2.3. Western Blot

Total cellular lysates were prepared by using RIPA lysis buffer. Proteins in cell lysates (50 *μ*g) were separated on 4–12% SDS-polyacrylamide minigels and electrotransferred to a PVDF membrane by iBlot Dry Blotting System (Invitrogen, Carlsbad, CA, USA). Antibodies used in this study were as follows: anti-Caspase 3, 8, and 9 antibodies (Cell Signaling Technology, Boston, MA, USA; 1 : 2000), anti-PARP antibody (Cell Signaling Technology, Boston, MA, USA; 1 : 2000), anti-BID antibody (Cell Signaling Technology, Boston, MA, USA; 1 : 2000), anti-FADD antibody (Cell Signaling Technology, Boston, MA, USA; 1 : 2000), anti-FLIP antibody (Cell Signaling Technology, Boston, MA, USA; 1 : 2000), antisurvivin antibody (Cell Signaling Technology, Boston, MA, USA; 1 : 2000), anti-Smac/Diablo antibody (Cell Signaling Technology, Boston, MA, USA; 1 : 10000), anti-Bad antibody (Santa Cruz Biotechnology, Santa Cruz, CA, USA; 1 : 1000), anti-Bax antibody (Santa Cruz Biotechnology, Santa Cruz, CA, USA; 1 : 1000), anti-DR4 antibody (Santa Cruz Biotechnology, Santa Cruz, CA, USA; 1 : 1000), anti-*α*-tubulin antibody (Santa Cruz Biotechnology, Santa Cruz, CA, USA; 1 : 20000), anti-DR5 antibody (ProSci Incorporated, San Diego, California, USA; 1 : 500), anti-Bcl-2 antibody (BD Biosciences PharMingen, San Diego, CA, USA; 1 : 1000), anti-XIAP antibody (BD Biosciences PharMingen, San Diego, CA, USA; 1 : 1000), anti-cIAP-1 antibody (R and D Systems, Minneapolis, MN, USA; 1 : 1000), anti-cIAP-2 antibody (R and D Systems, Minneapolis, MN, USA; 1 : 1000), anti-Mcl-1 antibody (Neomarker, Fremont, CA, USA; 1 : 1000), anti-Apaf-1 antibody (IMGENEX Corporation, San Diego, CA, USA; 1 : 2000), and anti-**β**-Actin antibody (Novus Biologicals Inc, Colorado, USA; 1 : 10000).

### 2.4. Viability Assay (MTT)

Cells were seeded at 10^4^ cells in 100 uL medium per well in   a 96-well plate and incubated (37°C, 5% CO_2_) overnight. Drugs of interest were added to each well and incubated (37°C, 5% CO_2_) for 2 days. MTT solution (5 mg/mL, Sigma Chemical Co., St. Louis, MO, USA) was added to each well at a final concentration of 0.5 mg/mL and incubated (37°C, 5% CO_2_) for 1-2 hours. Afterward, 100 uL of 10% SDS (Fluka, St. Louis, MO, USA) was added to each well and incubated at room temperature overnight.

Optical density at 570 nm was detected by SpectraMax M5 (Molecular Devices, Sunnyvale, CA, USA) for quantification.

### 2.5. Determination of ROS Production

ROS production was monitored by flow cytometry using DCFH-DA. This dye is a stable compound that readily diffuses into cells and is hydrolyzed by intracellular esterase to yield DCFH, which is trapped within cells. Thus, the fluorescence intensity is detected to quantify the amount of peroxide produced by the cells. To investigate the effect of periplocin and the combination of periplocin and TRAIL on generating intracellular ROS in HA22T/VGH, cells were pretreated with N-acetyl-cysteine (NAC) (30 mM) for 30 min and followed by periplocin (0.3, 0.03 *μ*M) alone or together with TRAIL (100 ng/mL). DCHF-DA (100 uM) were added to periplocin-treated cells with or without H_2_O_2_ (200 uM) for 1-2 hr. Green fluorescence was excited using an argon laser by flow cytometric analysis [21].

### 2.6. Quantification of Apoptosis by Annexin V/PI

After treated with periplocin alone or together with TRAIL for 24 hours, HA22T/VGH cells were washed and resuspended in the staining buffer and examined with the Vybrant Apoptosis Assay Kit (Invitrogen, Carlsbad, CA, USA) according to the manufacturer's instructions. The cell suspension was incubated with 2.5 *μ*L of Annexin V and 1 *μ*L of propidium iodide at room temperature for 15 min. The stained cells were analyzed by fluorescence activated cell sorter (FACS) analyses with a FACSCalibur flow cytometer, and data were analyzed using CellQuest software (BD Biosciences, San Jose, CA, USA).

### 2.7. Quantification of Apoptosis by Sub-G1 Peak

Trypsinized cells were washed with ice-cold PBS and fixed in 70% ethanol at −20°C for at least 1 h. After fixation, cells were washed twice, incubated in 0.5 mL of 0.5% Triton X-100/PBS at 37°C for 30 min with 1 mg/mL of RNase A, and stained with 0.5 mL of 50 mg/mL propidium iodide for 10 min. The fluorescence emitted from the propidium-DNA complex was quantitated by FACSCalibur flow cytometer (BD Biosciences, San Jose, CA, USA).

### 2.8. FACS Analysis

Cells were incubated with dye-labeled monoclonal antibodies (mAb) against target molecules for 30 min on ice. Stained cells were then washed twice and resuspended in cold buffer and analyzed with a FACScan flow cytometry (BD Biosciences, San Jose, CA, USA). More than 1 × 10^5^ cells were analyzed for each sample, and the results were processed by using WinMDI 2.8 software (Scripps Research Institute, La Jolla, CA, USA).

### 2.9. *In Vivo* Efficacy Study

All experimental protocols were approved by the Institutional Animal Care and Use Committee (IACUC number: ITRI-IACUC-2012-010M, Industrial Technology Research Institute of Taiwan, HsinChu, Taiwan. SCID (CB17/Icr-Prkdcscid/CrlBltw) mice were purchased from BioLASCO Ltd. (Ilan, Taiwan). Huh-7 cells (3 × 10^6^ cells per mice) in 100 *μ*L mix (equal volumes of PBS and Matrigel) were implanted subcutaneously (sc.) into the right flank of female SCID mice (6–8 weeks old). Tumor sizes were measured with calipers, and tumor volumes (mm^3^) were calculated with the following formula: *V* = *LS* ^2^/2 (where *L* is the longest diameter and *S* is the shortest diameter).

Huh-7 tumors were allowed to grow to 100–200 mm^3^. Periplocin (5–20 mg/kg; *n* = 6) or a vehicle control (*n* = 6) was intraperitoneally (IP) injected into tumor bearing mice once daily for 14 days. The formula of the vehicle is 10% NMP (M6762, Sigma-Aldrich, St. Louise, MO, USA), 20% Cremophor EL (C5135, Sigma-Aldrich, St. Louise, MO, USA), and 70% Saline. Tumor volume and body weight of animals were determined twice a week. The antitumor activity of treatments was illustrated by percentage of tumor growth inhibition (TGI). TGI was calculated as [1 − (tumor volume final − tumor volume initial for treated group)/(tumor volume final − tumor volume initial for vehicle group)] × 100.

### 2.10. Histology and Immunohistochemistry

At the end of the study, mice were sacrificed, and tumor samples were collected, fixed in formalin, and embedded in paraffin as tissue sections. Tissue sections were stained with hematoxylin and eosin (H and E) for general tissue morphology evaluation. The antihuman Ki67 antibody (1 : 500 dilution, IS-626, Dako, Glostrup Denmark) and antihuman cyclin-D1 antibody (1 : 500 dilution, IS-626, Dako, Glostrup, Denmark) were used in immunohistochemistry staining. Staining procedure was completed by using Autostainer Link 48 system (Dako, Glostrup Denmark). Five fields of every tumor sample were randomly selected, and the percentage of the Ki67-positive and cyclin-D1-positive cells was calculated to evaluate the proliferation of tumor samples.

## 3. Result

### 3.1. Periplocin as the Active Ingredient in Cortex Periplocae in Inhibiting the Growth of Hepatocellular Carcinoma Cells

**Figure 1:**
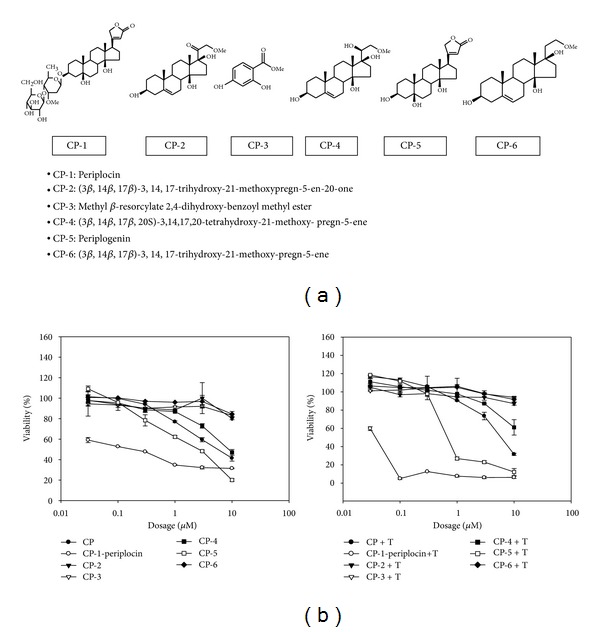
The effect of cortex periplocae extracts on HCC cell viability. (a) The chemical structures and names of purified cortex periplocae extracts are listed. (b) The cell viability of HA22T/VGH cells was determined by MTT assay after cells were treated with different concentrations of the indicated compound (0.03, 0.1, 0.3, 1, 3, and 10 *μ*M) with or without TRAIL (T) for 48 h.

Cortex periplocae (CP) is a traditional medicine capable of inhibiting cancer cell growth. To identify the active ingredients in CP that are responsible for its activity in inhibiting the growth of hepatocellular carcinoma (HCC) cells, we isolated and purified CP. After several rounds of purification, we found a group of compounds named CP-1 to 6 as major components in the fraction that can actively inhibit tumor cell growth (Figure 1(a)).

To further identify the active ingredients in the fraction, the pure compounds in the active fraction was examined one by one, and periplocin (CP-1) was identified as the most potent compound in inhibiting tumor cell growth with IC_50_ at 0.027 *μ*M (Figure 1(b)). The cotreatment of TRAIL and periplocin or periplogenin (CP-5) strongly enhances the growth inhibiting activity of periplocin and periplogenin (Figure 1(b)). Interestingly, periplocin is less toxic to normal cells. The cell viability of PBMC was more than 80% when treated with 300 *μ*g/mL periplocin (data not shown).

### 3.2. The Combination Treatments of Periplocin and TRAIL-Induced Apoptosis in TRAIL-Resistant HCC Cells

**Figure 2:**
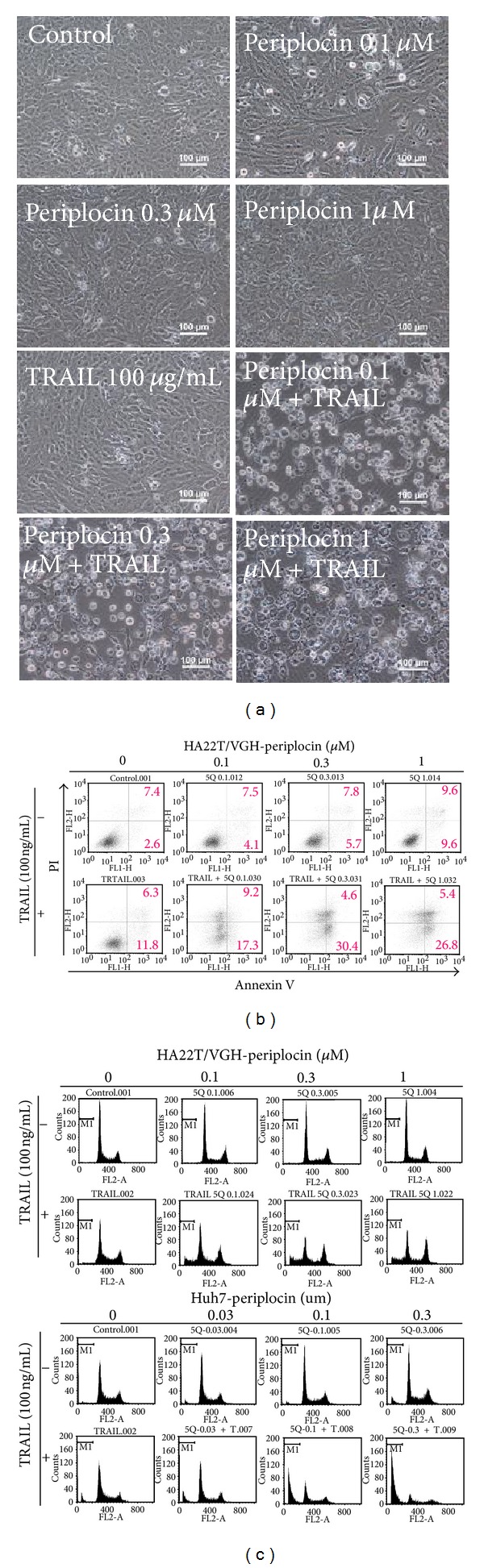
Periplocin and TRAIL treatments regulated cell apoptosis in HA22T/VGH cells. (a) HA22T/VGH cells were treated with periplocin alone or together with TRAIL for 24 h, and the morphology of cells was observed under microscope. (b) Cell apoptosis in HA22T/VGH cells regulated by periplocin and/or TRAIL was determined by FACS with Annexin-V-Fluorescein (*x*-axis) and PI (*y*-axis) double-staining system. (c) PI staining for both HA22T/VGH and Huh-7 cells after periplocin and/or TRAIL treatments. Apoptotic cells were quantified by FACS analysis (sub-G1 group).

Although TRAIL is a promising anticancer drug, more and more TRAIL-resistant cancers were reported. We sought to determine if periplocin can sensitize TRAIL-resistant HCC cells to TRAIL treatment. As shown in Figure 2(a), TRAIL or periplocin alone had little effect on the viability of HCC cells, but the combination of these two drugs showed cytotoxicity to TRAIL-resistant HCC cells.

To study if the combination treatments of periplocin and TRAIL sensitize TRAIL-resistant HCC cells and induce HCC apoptosis, HA22T/VGH cells were stained with Annexin V and PI to characterize cells in early and late stages of apoptotic processes accordingly. As shown in Figure 2(b), periplocin treatment increased the ratio of Annexin V and PI positive HCC cells. Cotreatment of TRAIL and periplocin further increased the ratio of Annexin V and PI positive cells. Therefore, TRAIL and periplocin synergistically induced cell apoptosis in HCC cells.

Moreover, the accumulation of cell debris after apoptosis was demonstrated by sub-G1 population in cell cycle analysis. Consistent with our previous results, periplocin dose dependently increased sub-G1 population in HCC cells, while the addition of TRAIL further increased the sub-G1 population in HCC cells (Figure 2(c)).

### 3.3. Periplocin and TRAIL Cotreatment Induces Apoptosis in HCC Cells by Inducing DR4 Expression and Activating Pro-Apoptotic Proteins

Intracellular reactive oxygen species (ROS) are involved in apoptotic pathways. Therefore, the effects of periplocin and/or TRAIL treatments on the intracellular ROS level in HA22T/VGH cells were examined. Although periplocin treatment alone or together with TRAIL induced intracellular ROS accumulation in HA22T/VGH cells, NAC pretreatment did not prevent cell apoptosis induced by TRAIL and periplocin cotreatment (supplemental Figure 1 (see Supplementary Material available online at http://dx.doi.org/10.1155/2013/958025.) and data not shown) 

**Figure 3:**
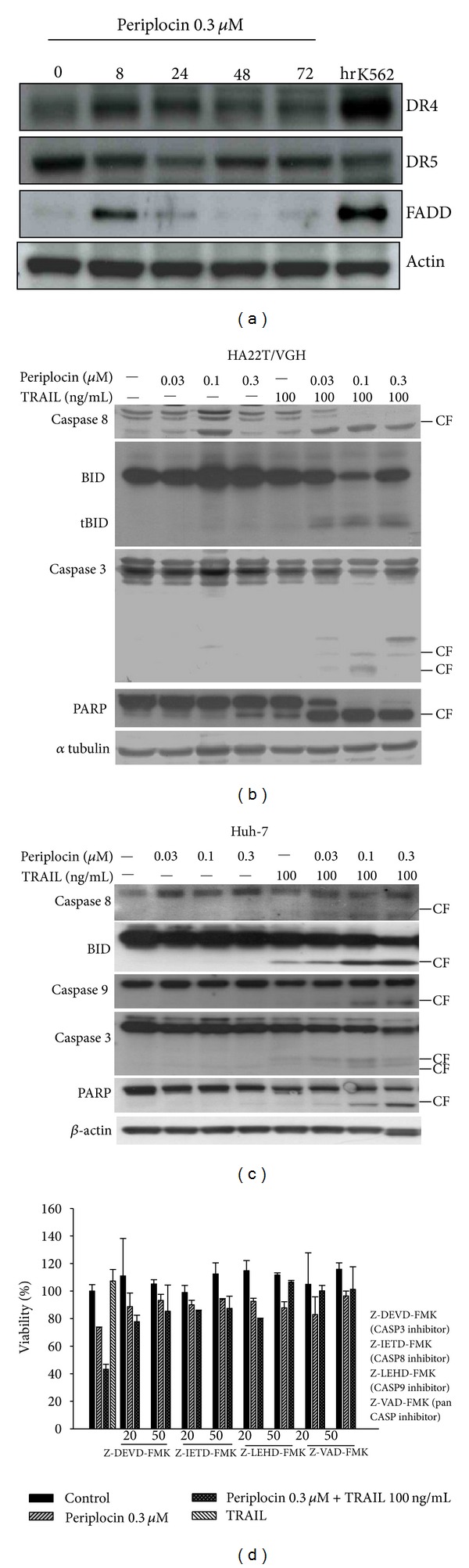
Treatments of periplocin and/or TRAIL activate DR4, FADD, and proapoptotic proteins in HCC cells. (a) The effect of periplocin treatment on the expression of DR4, DR5, and FADD was analyzed by western blot. (b) HA22T/VGH cells were treated with different doses of the indicated compounds for 24 h. Expressions of both proforms and cleaved forms of caspase-8, caspase 3, PARP, and BID were analyzed by western blotting. (c) Huh-7 cells were treated with different concentrations of the indicated compounds for 4 h or 24 h. The expression of caspase-8, caspase-9 was analyzed after indicated compounds treatment for 4 h, and the expression of caspase 3, PARP, BID was analyzed after the treatment of indicated compounds for 24 h. (d) HA22T/VGH cells were pretreated with 20 *μ*M or 50 *μ*M inhibitors against caspase-3 (Z-DEVD-FMK), caspasae-8 (Z-IETD-FMK), caspase-9 (Z-LEHD-FMK), and general caspase inhibitor (Z-VAD-FMK) for 3 hours prior to periplocin and/or TRAIL treatment. Cell viability was examined by MTT assay.

TRAIL induces cell apoptosis through interaction with death receptors DR4 and DR5 signaling. It was reported that compounds which upregulate DR4 and DR5 in HCC cells could sensitize TRAIL-resistant HCC cells to TRAIL treatment [22]. To investigate if periplocin sensitizes TRAIL-resistant cells through the same mechanism, the expression levels of DR4 and DR5 in HA22T/VGH cells with or without periplocin treatments were detected. Periplocin increased DR4 expression and further induced FADD expression in HA22T/VGH cells 8 hours after treatment. However, periplocin did not induce DR5 expression in HA22T/VGH cells (Figure 3(a)). The results were verified by FACS analysis (data not shown).

TRAIL binds DR4 and activates FADD. Activated FADD induces the cleavage of several proapoptotic proteins and activates them. We examined the activation of several apoptosis-related proteins, including the cleavage of BID, caspase 8, caspase 3, and PARP in HA22T/VGH. As expected, periplocin or TRAIL treatment alone had little effects on the cleavage of BID, caspase 8, caspase 3, and PARP. The combination of periplocin and TRAIL treatments strongly increase the cleavage of all these apoptosis-related proteins (Figure 3(b)). Similar results were observed in Huh7, another HCC cell line (Figure 3(c)).

To further confirm the importance of caspase activation in periplocin-regulated cell apoptosis with or without TRAIL treatment, caspase inhibitors were added to HA22T/VGH cells prior to periplocin and/or TRAIL treatments. Inhibitors against caspase 3, caspase 8, and caspase 9 partially rescued cell survival repressed by periplocin and/or TRAIL treatments, and pan inhibitor against all three caspases completely blocked cell death induced by periplocin and/or TRAIL treatments (Figure 3(d)).

### 3.4. Periplocin and TRAIL Cotreatment Induces Apoptosis in HA22T/VGH Cells by Inhibiting IAPs

**Figure 4:**
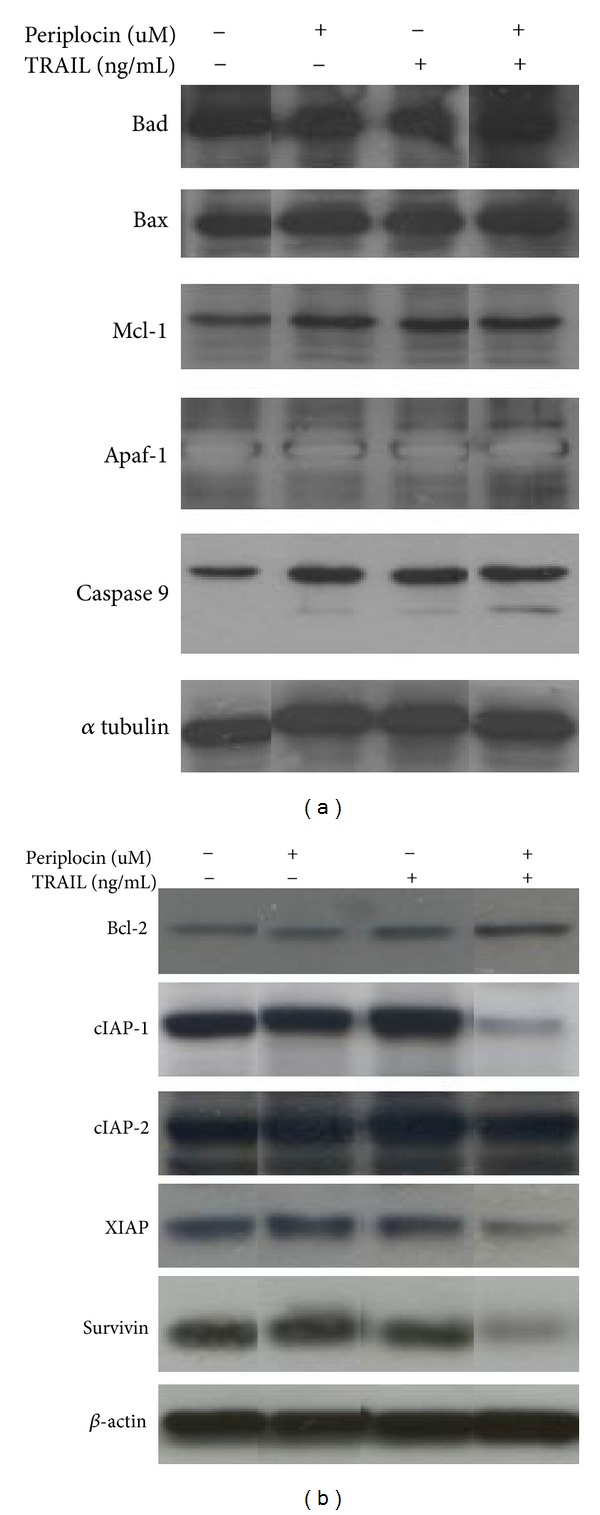
Cotreatment of periplocin and TRAIL activated IAP. (a) The expression levels of Bax, Bad, Mcl-1, apaf-1, and caspase 9 in HA22T/VGH in response to 1 *μ*M periplocin and/or 100 ng/mL TRAIL treatment were examined by western blot. (b) The expression levels of Bcl-2, cIAP-1, cIAP-2, XIAP, and survivin in HA22T/VGH in response to 1 *μ*M periplocin and/or 100 ng/mL TRAIL treatment were examined by western blot. The original blots are shown in  supplemental Figure 2.

Another reported mechanism for sensitizing TRAIL-resistant cell lines to TRAIL treatment is through regulating proteins involved in apoptotic pathways [23]. Since the cleavage of caspases can be induced either through intrinsic (mitochondrial mediated) or extrinsic (death receptor mediated) apoptotic pathways, the role of mitochondrial-mediated caspase cleavage was first investigated in HA22T/VGH with periplocin and TRAIL treatments. The expression levels of Bcl-2 protein family and several mitochondria dependent apoptotic regulators, including Bax, Bad, Mcl-1, and apaf-1, were detected before and after periplocin and/or TRAIL treatments. However, the treatments of periplocin and/or TRAIL did not affect the expression of apoptotic regulators (Figure 4(a) and supplemental Figure 2A). Nevertheless, the combination treatment of periplocin and TRAIL activated caspase 9 in two different HCC cell lines (Figures 3(c) and 4(a) and  supplemental Figure 2 ). Since the combination treatment of periplocin and TRAIL activated several caspases, we examined the effect of periplocin and TRAIL on members of inhibitors of apoptosis (IAP) family. The expression of several IAP family members, including cIAP-1, XIAP, and survivin, was repressed by the combination treatment of periplocin and TRAIL in HA22T/VGH cells (Figure 4(b) and  supplemental Figure 2 ).

### 3.5. Periplocin Represses Tumor Formation *In Vivo*

**Figure 5:**
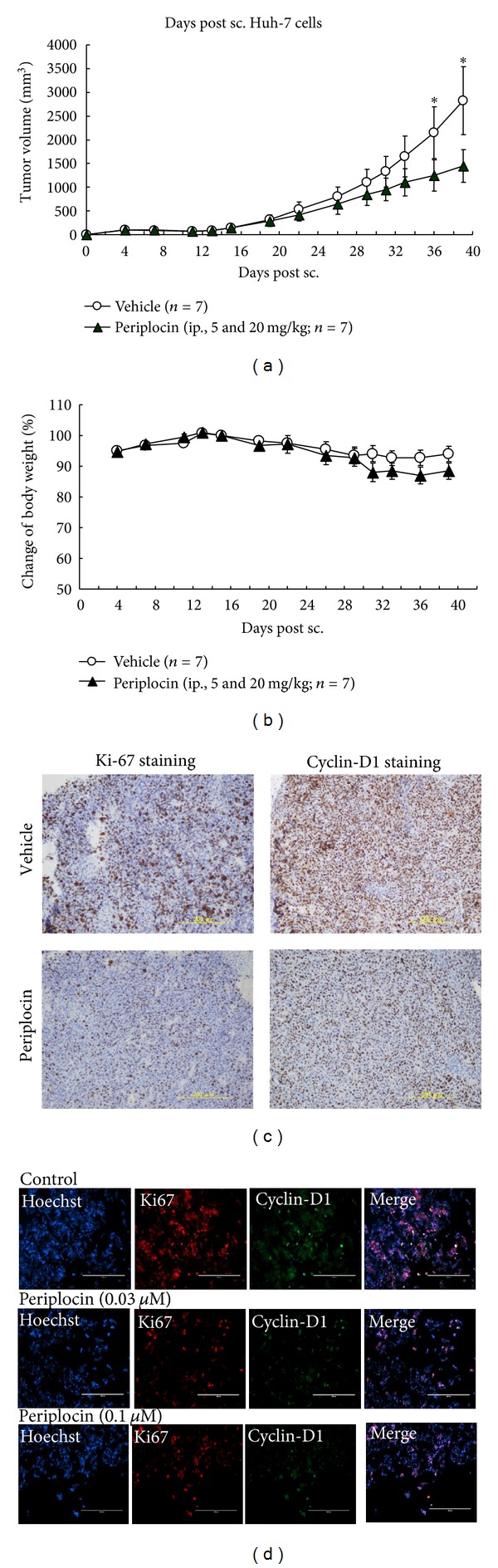
Periplocin treatments affected tumor growth in xenograft *in vivo *model. HCC cells were subcutaneously injected into SCID mice at 3 × 10^6^ cells. Periplocin was IP injected into SCID mice daily two weeks after initial tumor injection (5 mg/kg on day 15 to day 29 and 20 mg/kg on day 29 to day 35). (a) Tumor size was measured every 2–4 days throughout the study (*P < 0.05). (b) Body weight of control and treated mice were measured every 2–4 days throughout the study. (c) After the mice were sacrificed, tissue sections were prepared and stained with anti-Ki67 and anticyclin-D1 antibodies to evaluate cell proliferation. (d) Huh-7 cells were fixed, and immunofluorescent stained by Hoechst 33258 (blue staining), Ki67 (red staining), and cyclin-D1 (green staining).

In addition to* in vitro* mechanistic studies, we also verified the potency of TRAIL and periplocin on repressing tumor growth* in vivo*. Since TRAIL was expressed in NK cells in mice, we treated tumor-bearing mice with only periplocin in this *in vivo* study [24, 25]. To test the antitumor activity of periplocin *in vivo*, HCC cells were subcutaneously injected into SCID mice, and periplocin was intraperitoneally (IP) injected daily two weeks after the initial injection of tumor cells. Since the mice were well-tolerant to 5 mg/kg periplocin after 2 weeks of treatment, the dose of periplocin were raised to 20 mg/kg daily throughout the study. Periplocin was able to inhibit HCC growth in xenograft model. Tumor growth inhibition (TGI, %) of periplocin treatment was 51 ± 11% after 24 days treatment, (Figure 5(a)). When treated with periplocin, the mice body weight was slightly decreased at the dose of 20 mg/kg when compared to the vehicle group. However, the body weight kept at around 90 percent of control group, and no further body weight loss was observed (Figure 5(b)). These data showed that periplocin strongly inhibited tumor growth of Huh-7 tumors without obvious side effects.

In Ki67 immunochemistry analysis, periplocin could inhibit the Ki67 expression in tumor samples (Figure 5(c)). After selecting five random fields and calculating the Ki67 positive cells of every tumor sample, the percentage of Ki67-positive cells in vehicle group was 57.3 ± 0.67%. The percentage of Ki67-positive cells in periplocin-treated group was 22.78 ± 10.09%. The result showed that periplocin could significantly inhibit the Ki67 expression in Huh-7 tumors, which suggested that periplocin inhibited tumor growth *in vivo*.

To further verify the periplocin-inhibited tumor growth* in vivo*, we also examined the expression of cyclin-D1 in Huh-7 tumors. In cyclin-D1 immunochemistry analysis, the percentage of cyclin-D1-positive cells in vehicle group was 76.87 ± 2.93%. The percentage of cyclin-D1-positive cells in periplocin-treated group was 58.85 ± 5.05%. The result showed that periplocin could inhibit the cyclin-D1 expression in tumor samples (Figure 5(c)). To verify the role of periplocin in cyclin-D1 expression, Huh-7 cells were seeded on Millipore Millicell EZ slide (2 × 10e4/well) for 24 h and subsequently treated with different concentrations of periplocin for 24 h. As expected, periplocin dose-dependently repressed cyclin-D1 expression in Huh-7 cells (Figure 5(d)).

## 4. Discussion

Periplocin is a cardiac glycoside structurally similar to digoxin. The pharmacological function of periplocin is also similar to digoxin and has been used to treat heart diseases. Nevertheless, digoxin was shown to block cancer growth through inhibiting HIF-1*α* signaling pathway in cancer cells [26]. Indeed, *Digitalis* was used as treatment for breast cancer patients and reduced cancer recurrence rate [27, 28]. Compounds structurally similar to digoxin also possess antitumor activity [29]. Several studies suggest that cardiac glycoside induces cell apoptosis. Digoxin induces apoptosis through activating Cdk5 [30]. In addition, *Digitalis* was reported to induce mitochondria-dependent apoptotic pathways in guinea-pig cardiomyocytes [31].

TRAIL induces cell apoptosis via DR4 and DR5, activates Fas-associated death domain (FADD) and caspase 8, and signals through both mitochondria-dependent and -independent pathways [32]. There are three antagonistic decoy receptors DcR1, DcR2, and osteoprotegerin that interact with TRAIL but cannot transmit apoptotic signal. Therefore, cancer cells could gain TRAIL-resistance by overexpressing DcR1, DcR2, or osteoprotegerin [33–35]. In addition, defects in adaptor protein FADD or caspase 8 can also lead to TRAIL resistance [36, 37]. In this study, we demonstrated that periplocin induced DR4 and FADD expression in TRAIL-resistant HCC cells. The combination treatment of TRAIL and periplocin activated caspase 8, the key caspase for both mitochondria-dependent and-independent apoptotic signaling pathways, in TRAIL resistant HCC cells.

The combination treatment of periplocin and TRAIL induced HCC cell apoptosis through activating IAP. IAPs are members of a protein family that regulate apoptosis. Currently there are 8 known members in this family, and X-linked inhibitor of apoptosis protein (XIAP) is the best characterized member in the family. XIAP blocks apoptosis by binding and inactivating caspase 3, 7, and 9 [38]. Other IAP, including c-IAPl, c-IAP2, and survivin, have been shown to bind to caspase 3 and 7 [39]. Indeed, we observed the repression of XIAP, c-IAP1, and survivin, and the deactivation of caspase 3 and 9 by TRAIL and periplocin treatments in this study.

The expression of TRAIL by NK cells was demonstrated both* in vitro* and* in vivo* [24, 25, 40, 41]. We took advantage of that and designed an experiment that combined endogenous TRAIL and exogenous periplocin treatment. The injected periplocin was able to repress HCC tumor growth* in vivo*.

While purifying cortex periplocae, we identified six compounds with potential antitumor activity (Figure 1). Among these compounds, periplocin (CP-1) and periplogenin (CP-5) are the two compounds with cytotoxicity against cancer cells, and periplocin is more potent than periplogenin. Structurally, periplocin is different from periplogenin by only one disaccharide residue. It was reported that saccharide residues are involved in the recognition of plant root surfaces by zoospores [42]. Therefore, it is not surprising that saccharide residues can affect protein interactions. However, detailed mechanisms of how periplocin interacts with cell surface molecules and how does the disaccharide residue regulate cell apoptosis require further investigation.

In traditional herb medicine, patients are usually treated with multiple raw materials. Sometimes it is hard to purify one single active ingredient since multiple components are required to achieve therapeutic goals. In this study, we purified an active ingredient, periplocin, with cytotoxicity against HCC cells from cortex periplocae. Interestingly, maximum cytotoxicity against TRAIL-resistant HCC was achieved by combining the treatment of periplocin and TRAIL. The idea of the combination treatment is consistent with the concept of traditional medicine. Provided the mechanistic studies, the combination treatments of active ingredients from herb medicine and chemical synthesized compounds or protein drugs could be potential treatment options for drug-resistant cancers.

## 5. Conclusion

**Figure 6:**
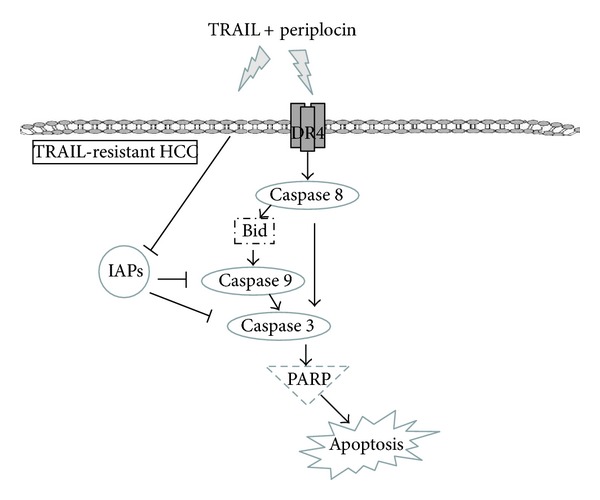
Mechanisms of resensitizing TRAIL-resistant HCC cells by periplocin. Periplocin can resensitize TRAIL resistant HCC by inducing the expression of DR4. Therefore, the combination treatments of TRAIL and periplocin can induce cell apoptosis through direct activation of caspase signaling and indirect inhibition of IAPs.

In this study, we demonstrated that periplocin could sensitize TRAIL-resistant HCC to TRAIL treatment, and the combination treatment of TRAIL and periplocin can induce apoptosis in TRAIL-resistant HCC. Furthermore, we showed that periplocin sensitized TRAIL-resistant HCC cell lines to TRAIL through the following two mechanisms. First, periplocin induced the expression of DR4 and FADD to activate proapoptotic signaling pathways. Second, the cotreatment of TRAIL and periplocin suppressed several IAP, which also led to the activation of proapoptotic signaling pathways. Our working model is shown in Figure 6. Further studies are required to apply periplocin clinically.

## Supplementary Material

Supplemental Figure 1: The ROS production in HA22T/VGH cells was regulated by periplocin and/or TRAIL treatment. HA22T/VGH cells were treated with periplocin and/or TRAIL for 1 hr with or without 1-hr pretreatment of NAC (30 mM). DCHF-DA was added into each sample for 30 min, and the DCF fluorescence intensity in cells was detected by FACS analysis.
Supplemental Figure 2: The dose-dependent effect of periplocin on apoptosis-related proteins. (A) The expression levels of Bax, Bad, Mcl-1, apaf-1, and caspase 9 in HA22T/VGH in response to different doses of periplocin treatments with or without TRAIL treatment were examined by Western blot. (B) The expression levels of Bcl-2, cIAP-1, cIAP-2, XIAP, and survivin in HA22T/VGH in response to different doses of periplocin treatments with or without TRAIL treatment were examined by Western blot.
