# Supplementary figures and images for: Antitumor Effect of Periplocin in TRAIL-Resistant Human Hepatocellular Carcinoma Cells through Downregulation of IAPs
Source: Evid Based Complement Alternat Med. 2013 Jan 1;2013:958025. doi: 10.1155/2013/958025 (PMC3549389; doi:10.1155/2013/958025)

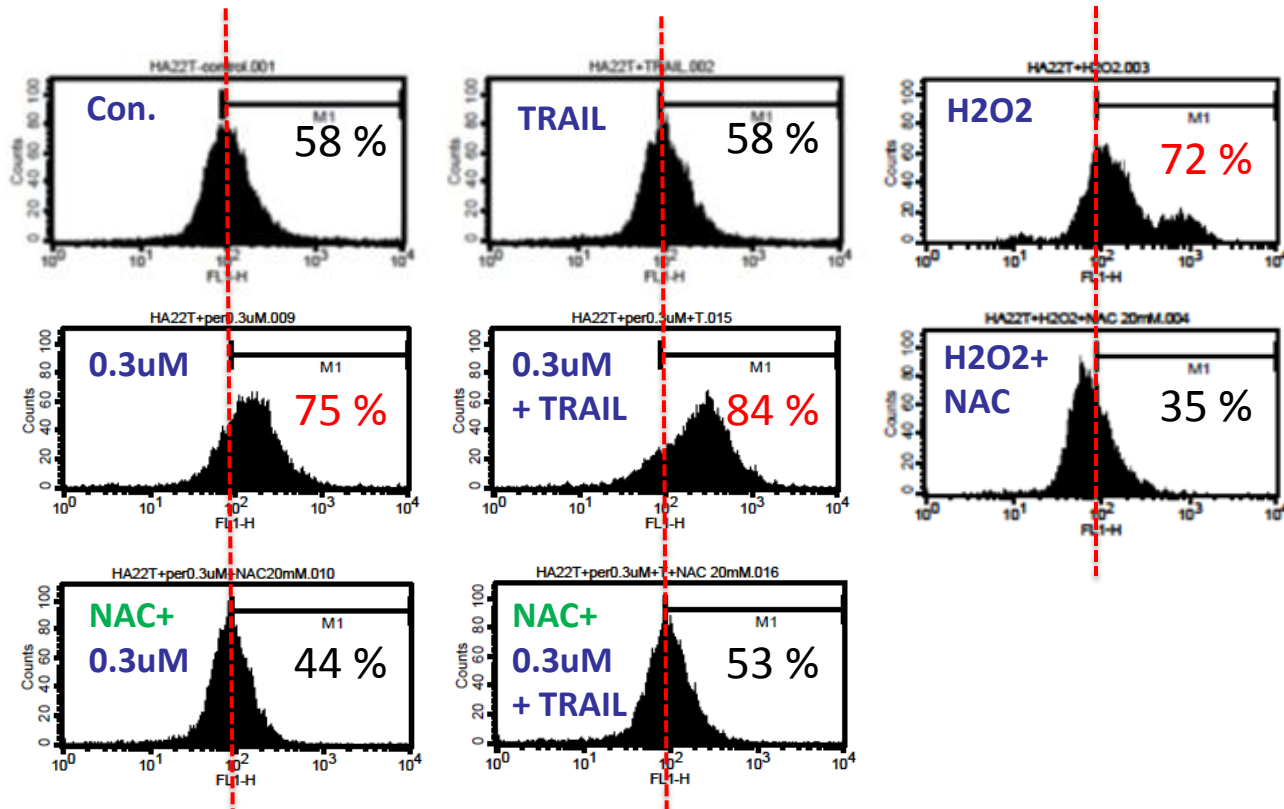

**A.**

| Periplocin (uM) | - | 0.1 | 0.3 | 1 | -   | 0.1 | 0.3 | 1   |
|-----------------|---|-----|-----|---|-----|-----|-----|-----|
| TRAIL(ng/ml)    | - | -   | -   | - | 100 | 100 | 100 | 100 |

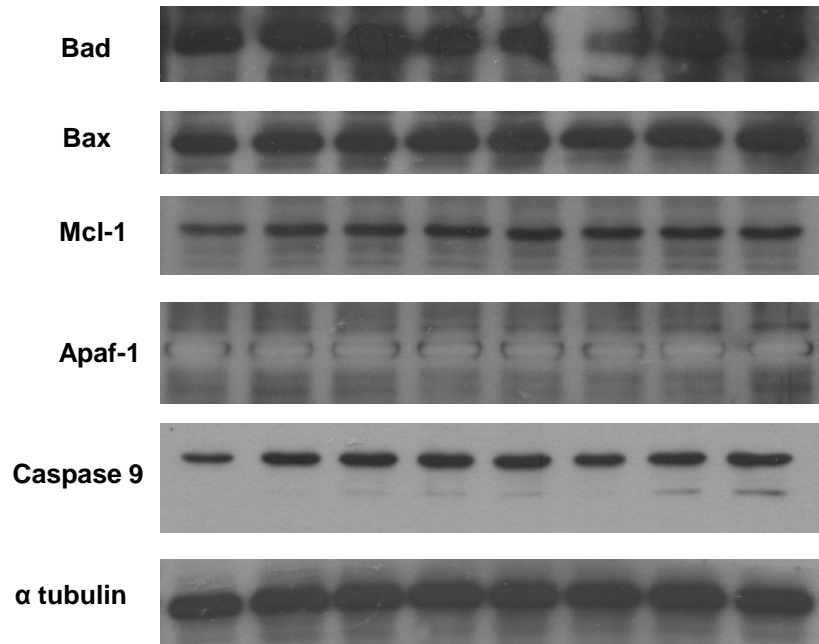

**B.**

| perplocin (uM) | - | 0.1 | 0.3 | 1 | -   | 0.1 | 0.3 | 1   |
|----------------|---|-----|-----|---|-----|-----|-----|-----|
| TRAIL(ng/ml)   | - | -   | -   | - | 100 | 100 | 100 | 100 |

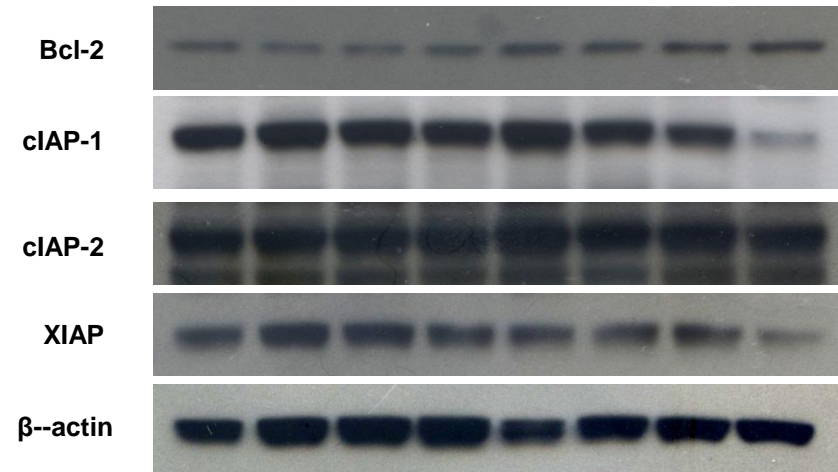

| perplocin (uM) | - | 0.3 | 1 | 3 | -   | 0.3 | 1   | 3   |
|----------------|---|-----|---|---|-----|-----|-----|-----|
| TRAIL(ng/ml)   | - | -   | - | - | 100 | 100 | 100 | 100 |

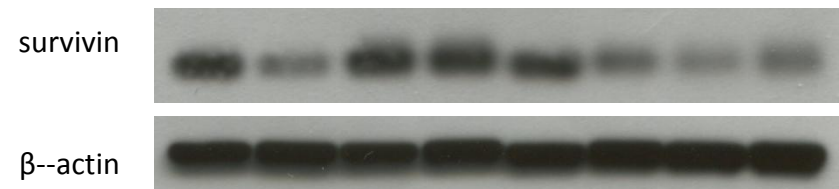

Supplement: Supplementary file 1 — Supplemental Figure 1: The ROS production in HA22T/VGH cells was regulated by periplocin and/or TRAIL treatment. HA22T/VGH cells were treated with periplocin and/or TRAIL for 1 hr with or without 1-hr pretreatment of NAC (30 mM). DCHF-DA was added into each sample for 30 min, and the DCF fluorescence intensity in cells was detected by FACS analysis. Supplemental Figure 2: The dose-dependent effect of periplocin on apoptosis-related proteins. (A) The expression levels of Bax, Bad, Mcl-1, apaf-1, and caspase 9 in HA22T/VGH in response to different doses of periplocin treatments with or without TRAIL treatment were examined by Western blot. (B) The expression levels of Bcl-2, cIAP-1, cIAP-2, XIAP, and survivin in HA22T/VGH in response to different doses of periplocin treatments with or without TRAIL treatment were examined by Western blot. [file 958025.f1.pdf]
